# Blueprints for the Next Generation of Bioinspired and Biomimetic Mineralised Composites for Bone Regeneration

**DOI:** 10.3390/md16080288

**Published:** 2018-08-20

**Authors:** Pamela J. Walsh, Kathryn Fee, Susan A. Clarke, Matthew L. Julius, Fraser J. Buchanan

**Affiliations:** 1 School of Chemistry and Chemical Engineering, Queen’s University Belfast, David Keir Building, Stranmillis Road, Belfast BT9 5AG, UK; 2 School of Mechanical and Aerospace Engineering, Queen’s University Belfast, Ashby Building, Stranmillis Road, Belfast BT9 5AH, UK; k.fee@qub.ac.uk (K.F.); F.Buchanan@qub.ac.uk (F.J.B.); 3 School of Nursing and Midwifery, Queen’s University Belfast, MBC, 97 Lisburn Road, Belfast BT9 7BL, UK; s.a.clarke@qub.ac.uk; 4 Department of Biological Sciences, St. Cloud State University, St. Cloud, MN 56301, USA; mljulius@stcloudstate.edu

**Keywords:** coccolithophore, biomineralisation, bone tissue engineering

## Abstract

Coccolithophores are unicellular marine phytoplankton, which produce intricate, tightly regulated, exoskeleton calcite structures. The formation of biogenic calcite occurs either intracellularly, forming ‘wheel-like’ calcite plates, or extracellularly, forming ‘tiled-like’ plates known as coccoliths. Secreted coccoliths then self-assemble into multiple layers to form the coccosphere, creating a protective wall around the organism. The cell wall hosts a variety of unique species-specific inorganic morphologies that cannot be replicated synthetically. Although biomineralisation has been extensively studied, it is still not fully understood. It is becoming more apparent that biologically controlled mineralisation is still an elusive goal. A key question to address is how nature goes from basic building blocks to the ultrafine, highly organised structures found in coccolithophores. A better understanding of coccolithophore biomineralisation will offer new insight into biomimetic and bioinspired synthesis of advanced, functionalised materials for bone tissue regeneration. The purpose of this review is to spark new interest in biomineralisation and gain new insight into coccolithophores from a material science perspective, drawing on existing knowledge from taxonomists, geologists, palaeontologists and phycologists.

## 1. Introduction

The biomineralisation of marine organisms involves a multi-stage process which has evolved multiple times over 600 million years. The mineral synthesis by these organisms occurs mostly under ambient temperatures using chemical compounds, which are found in abundance in their natural environment. Although only limited precursors (e.g., calcium or magnesium ions) are involved, over 70 biogenic minerals can be formed [1,2]. Often, material attributes such as chemical composition and crystallinity of the initial precipitate differ significantly from the final mineral formed. Mature biogenic minerals offer a host of unusual nanoscale morphologies, with nanoparticles/spheres forming crystals [3]; these are reinforced by an organic matrix, resulting in surprising mechanical properties [4]. At a nano-/microstructural level, marine biocomposites differ considerably in terms of their mechanical strength from those of their bioinspired, synthetic materials counterparts [5]. For example, their fracture toughness exceeds that of synthetic minerals by two to three orders of magnitude, despite being highly mineralised (>95%) [6]. Synthetic biomimetic materials often lack hierarchical organisation and structures, particularly at a nano level, which is considered an essential component for enhanced biological materials [4]. Understanding the macromolecular interaction between organic and inorganic compounds, and the biochemical pathways in specific calcified marine organisms (crustaceans [4] corals [7], shells [6,8], macro- [7,9] and micro-algae [10]) are important in unravelling their complex hierarchical structures. Strategic placement of organics (<5%) within the matrices is one of several factors thought to be critical to their superior mechanical properties [6]. More detailed studies of marine organisms are required to understand crystallochemical mechanisms [2], hierarchical structure and polymer chemistry of the organic template that initiates nucleation [1], particularly in coccolithophores [11]. Although biomineralisation has been studied for decades, few studies have investigated in situ crystal growth formation in calcified marine organisms [3].

Coccolithophores, dinoflagellates and diatoms are three major eukaryotic primary phytoplankton producers that play an important role in carbon and nutrient cycling in the oceans. Their ability to evolve independently has contributed to their successful survival, despite competitive interactions for dissolved Si ions. Until recently, dissolved Si ions were considered essential in the two primary forms of biomineralisation: (1) silicification (diatoms) and (2) calcification (coccolithophores, some dinoflagellates (a single family)). However, a recent study by Durak et al. has reported that Noelaerhabdaceae and Pleurochrysidaceae coccolithophore families have evolved to mineralise without silica [12]. Calcification in coccolithophores [13] is a distinctly different process to silicification in diatoms [14]. The cellular processes that underpin both these mechanisms remain poorly understood. Coccolithophores have an equally diverse range of unique morphologies to that of diatoms, which have been extensively studied by both taxonomists [15] and material scientists [16]. However, they have sparked significantly less interest in the field of material science, despite their unique morphologies, comprehensively reviewed by Young et al. [17].

**Figure 1:**
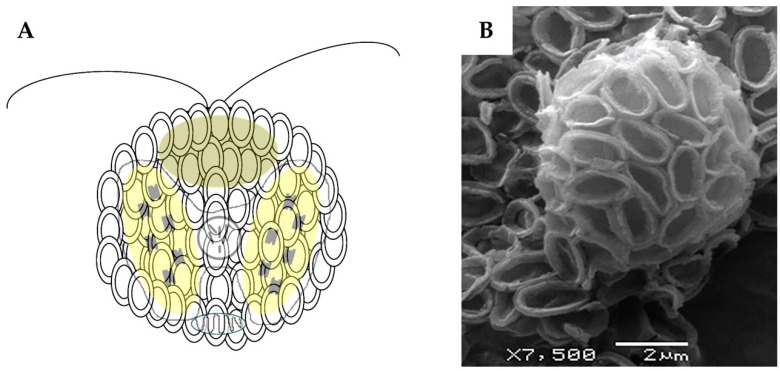
(**A**) Schematic and (**B**) Scanning Electron Microscope (SEM) micrograph of calcite coccosphere (cell wall containing coccoliths fused together) of *Pleurochrysis carterae* CCMP647.

Diatoms and coccolithophores are tightly regulated by their innate biological mechanisms, which determine their size and shape, resulting in unique, intricate architectures. One unusual characteristic of coccolithophores are the tiny calcite platelets that fuse together to form their outer cell wall, known as the coccosphere (Figure 1).

The scope of marine organisms in the development of new materials is endless, with applications in many fields beyond biomaterials, e.g., catalysts to sensors [5,16]. Other marine lifeforms such as plants, arthropods and mammals [18] also offer unique templates; however, they are beyond the scope of this review. In terms of biomaterials, marine organisms have provided inspiration for the development of new functional biomaterials by either mimicking or directly converting marine materials into an alternative inorganic material. Both strategies have been attempted with nacre [19,20], coral [7], sea urchin [21] and crustaceans [4]. This review focuses on mineralised marine organisms with particular emphasis on heterococcoliths and their potential biomedical applications. They differ from other marine calcifiers, as the coccoliths that form the external coccospheres mineralise intracellularly prior to being secreted to the exterior face of the exoskeleton cell wall [22]. The mineralisation process, which occurs inside an intracellular compartment, is still poorly understood [11], but is responsible for the precise control of the coccolith morphology [22]. Their complicated, ornate, hierarchical structure starts on a nanometer scale, and is formed from a collection of specialised proteins/genes that are regulated by Ca^2+^ ion environmental flux and cell cycle progression [23,24]. Across different species, coccosphere architecture can vary markedly; however, within the same species, it is replicated precisely from generation-to-generation. Several studies have investigated biomineralisation in coccolithophores, focusing on their organic template, ion transporters and macromolecular structures [10,25]. However, a detailed knowledge of the process still remains frustratingly incomplete, particularly on a subcellular level, e.g., two amino acids associated with the baseplate of *Pleurochrysis carterae* (*P. carterae*) remain unidentified [26], which hampers their translation into biomimetic biomaterials [25]. Significant challenges still remain on material characterisation [27], ‘omics’ profiling [25] and biological function of coccolithophores, despite the availability of advanced molecular and genetic approaches.

## 2. Coccolithophores

**Figure 2:**
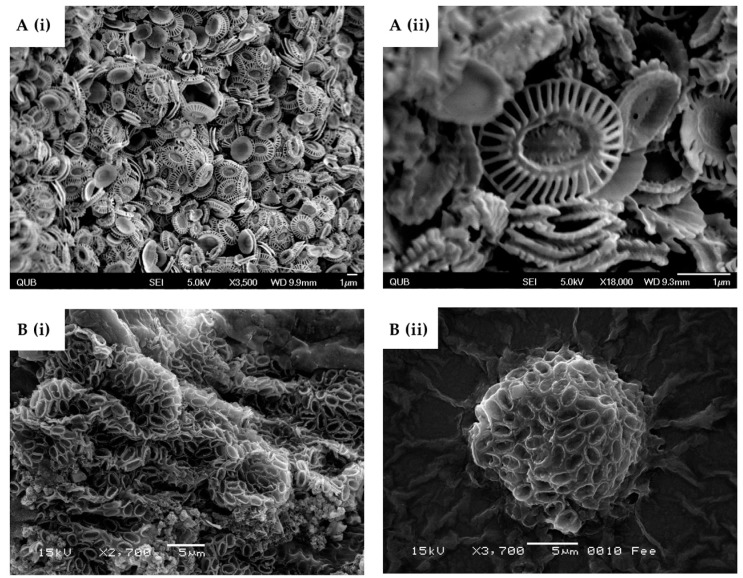
SEM Micrographs of (**A**) *E. huxleyi* CCMP 1516 (i) ×3500 (ii) ×18,000 (**B**) *P. carterae* CCMP647 (i) ×2700 (ii) ×3700.

Despite their miniature size (<50 μm), coccolithophores have the ability to synthesise intricate, inorganic structures (Figure 2) with equal complexity and function in their hierarchical ordering on a nano- to micro-scale [1,28]. There are two types of coccoliths. The first are heterococcoliths, which consist of plate-like wheels, extended spicules or elaborate coronets connected by a tube. These are much more complex-shaped structures [29,30] than the second type, holococcoliths, which form a rhombohedral shape with a simple tessellate crystal structure [30,31]. The distinctly different microstructures between heterococcoliths and holococcoliths would suggest a significantly different mechanism of biomineralisation [32] and considerable physiological diversity [12]. Despite being more complex with a more diverse biological profile, the most commonly studied coccolithophores are *Emiliania huxleyi* (*E. huxleyi*) (Figure 2A) and *P. carterae* (Figure 2B), which are both heterococcoliths [31,33]. The literature has a strong bias (>82% of database entries [34]) towards the study of *E. huxleyi*, which belongs to the Noelarhabdaceae family. In modern oceans, *E. huxleyi* and *Gephyrocapsa oceanica* are the two most abundant coccolithophore species present [12]. The *E. huxleyi* strain CCMP1516 has evolved into a model system for physiology, molecular, genomics and environmental studies [35], with a complete genome sequence available [36]. Most of the work dating from before 2009 has no specific reference to the strain of *E. huxleyi* studied, which has almost certainly resulted in data anomalies. Langer et al.’s study into intra-strain variation found one in four strains tested varied in coccolith formation when subjected to the same stimuli [37]. This highlights the importance of studies using specific strains to understand biomineralisation. Several environmental studies that use coccolithophores’ biomineralisation as a marker have reported conflicting results [35], to which intra-strain variation may be a contributing factor. Strain anomalies could potentially mean that many observations and results are not simply conflicting, but fundamentally incorrect.

Heterococcoliths consist of an organised array of coccoliths that are secreted through the plasmalemma to the outer surface of the cell. This does not occur until the coccolith is fully formed inside the golgi-derived coccolith vacuole, which is located in an intercellular compartment [10]. The necessary force required to secrete coccoliths to the outer surface may be generated from actin and microtubule polymerisation [38]. Mature coccoliths form a coccosphere array on the extracellular surface surrounding the coccolith vacuole. Growth conditions [39] and phylogeny [40] will influence the coccolith size, but in general they have a mean diameter of 4 μm [39]. Each coccolith is replicated precisely and is uniform within the species, with an exceptional ability to be controlled in both *c*-axis and *a*-axis orientation [41] in a highly reproducible manner. This creates a distinctive pattern with two interlocking units in vertical and radial directions. The algorithm produced is described by Young et al. as the Vertical/Radial (V/R) growth model based on observations in *E. huxleyi* [31] and later validated in other heterococcolith species [41]. After being secreted by the cell, the coccolith plates form arrays known as coccospheres. The core coccoliths create a spine that acts as a template to guide the outer crystalline units [31]. Coccospheres typically contain a monolayer of approximately 12 to 15 coccoliths [39], which grow into a multi-layer cell wall. Under controlled lab conditions, *E. huxleyi* produces coccoliths continuously at a rate of 1 coccolith plate every 3 h [42]. In their natural environment, smaller species have been cited to contain an average of 22 coccoliths, whereas larger species can contain up to 72 [40]. Most literature on coccolithophore geometry is based on *E. huxleyi,* which has been reported to have a mean coccolith diameter of 3.5 μm, which form into a coccosphere of 6.9 μm [39,40]. Often the number of coccoliths forming the sphere is limited by the growth environment [39].

## 3. Coccolithophore Organics

Classic biomineralisation assumes an organic substrate, anchored by calcite crystals that continue to participate in inorganic epitaxial layer formation. With the advent of sophisticated analytical techniques, new light has been shed on the role of organics. Initially, the organics were considered to act as a glue, providing an interfacial toughening mechanism [4]. Further studies revealed that the role of organics was not limited just to interfacial toughening, but was also critical in crystallisation, growth and the mechanical strengthening of the composite [4,19]. In the early 90s, Young et al. suggested that it was the acidic polysaccharide-binding template of coccolithophores that mediated their crystallographic orientation and growth [31]. This was supported by a more recent study by Gal et al. who reported that soluble, negatively charged polysaccharides were an essential foundation for calcite crystals to form in the correct location [26]. Three acidic polysaccharides, termed PS-1, -2 and -3, have been identified in coccolith plates of *P. carterae* [13]. These biomacromolecules are hypothesised to be synthesised in the golgi (the coccolith vesicle), forming the template [10,24]. The golgi contains fluid isolated from the cytoplasm [30]. The cytoplasm is of key importance in the understanding of cellular function; however, its biochemistry is often overlooked [43]. The coccolith template has a strong affinity for divalent cations (e.g., Ca^2+^ and Mg^2+^ ions) due to the acidic nature of the polysaccharides [44], and has thus been implicated in facilitating nucleation and subsequent crystal growth. This was considered adequate to regulate crystal growth and morphology when combined with two interlocking growth cycles in a particular direction under cytoskeletal control [28]. Yang et al. suggests nucleation is promoted through binding strength as a result of the interaction between polysaccharides and calcite surfaces [45]. In their research, they used *E. huxleyi* as a model species, and observed that binding to polar surfaces was dependent on polysaccharide termination, thus controlling calcite growth. While other groups have reported that stereochemistry controlled calcite nucleation in both *P. carterae* and *E. huxleyi* [46,47]. In marine invertebrates e.g., lobsters, acidic polysaccharides with carboxyl and sulfate functional groups have been shown to orientate CaCO_3_ nucleation [48]. In general, there is strong qualitative evidence in the literature that indicates the chemical structure of polysaccharides contributes to controlling biomineralisation processes [26,45,47].

Earlier work by Marsh et al. on *P. carterae* characterised the chemistry of PS-1 and PS-3 to be mainly galacturonic acid, whereas PS-2 was found to have the more unique polymer structure of a disaccharide unit [49]. These calcifying macromolecules were also thought to contain glucuronic acid and its oxidative by-product at the C2-C3 bond (meso-tartaric acid and glyoxylic acid) [50]. The calcium-binding capacity of PS-2 is the highest of any known mineral-associated polyanion, with a net ionic charge of -4 per repeating unit [49]. The organic coating around the coccolithospheres, combined PS-1 and PS-3 polysaccharides, whereas PS-2 forms the organic baseplate template at the interface of calcite crystals. Less attention has been given to the role of PS-3, which is thought to function during the crystal growth phase of mineralisation [51]. In *E. huxleyi*, mineralisation is associated with polysaccharides in the labyrinthine system (reticular body) [10], whereas in *P. carterae* a large number of 20 nm particles, known as coccolithosomes [30], are found to agglomerate around the rim of the baseplate [26,49]. The labyrinthine system in *E. huxleyi* is formed at the distal surface of the coccolith vesicle and is present throughout nucleation [49]. PS-2 (or any analogous polyanion) found in *P. carterae*, however, is not expressed in *E. huxleyi* [49]. The monosaccharide units that have been identified in the baseplate of *E. huxleyi* include ribose, xylose, mannose and glucose [45]. Not all polysaccharides identified in coccolithophores will play a role in calcite nucleation. It is likely that some will provide an inert scaffolding support to proteins, similar to other marine organisms, e.g., chitin in mollusk shells [47].

Several groups are currently attempting to isolate and identify all biomacromolecules present in coccolithophores with particular emphasis on the precursor polysaccharides and proteins used in the baseplate template that produces coccoliths. Although polysaccharides and proteins have similar attributes, e.g., acidic nature, their structure and function is significantly different [50]. In marine organisms, only a few biomineralisation proteins have been identified to date. The proteins identified are generally small acidic proteins or glycoproteins often rich in glutamic or aspartic acid [33], that have the ability to bind Ca^2+^ reversibility [52]. Most of the proteomic literature on coccolithophores to date only identifies one protein, the calcium-binding glycoprotein “GPA”, which is intrinsically associated with coccolith polysaccharides, and is thought to play a significant role in biomineralisation [23,33,53]. Gal et al. recently reported unidentified amino acids present in the soluble fraction of the coccolith baseplate (*P. carterae*), these have also been reported in other studies, which also failed to identify them [26]. However, Gal et al. went a step further to show they are involved in the biomineralisation process. They isolated the organic fraction of the baseplate, solubilised it, and then used it to nucleate calcite precursors in vitro to synthesise coccolith baseplates. Sakurada S et al.’s [54] work supports Gal’s findings, suggesting that proteins associated with PS-2 in the baseplate initiate biomineralisation in coccolith formation in *Pleurochrysis haptonemofera*. Quinn et al., reported that GPA was expressed in both the mineralised and non-mineralised cells of *E. huxleyi* [23], this observation is also supported by a study by Kegel et al. [55]. Both studies found no evidence of GPA in mineralised *E. huxleyi* CCMP1516, suggesting gene regulation at a transcript level, or that it is specific to their non-mineralisation life cycle [55].

Coccolithophores alternate between complex mineralised diploid cells and non-mineralised haploid cells during the haplo-diplontic life cycle [35,56]. Natural populations tend to be dominated by mineralised diploid cells [56]. Thus, in theory, by comparing the RNA of mineralised diploid cells to non-mineralised haploid cells, it should thus be possible to identify those genes responsible for biomineralisation. However, in reality, proteins associated with biomineralisation that have been identified in other mineralised marine organisms (e.g., MSP130 found in sea urchins) have also been detected in non-mineralised organisms (e.g., entoprocta [57]). This would suggest that these proteins might have more than one function. Dassow et al. speculated that the GPA might perhaps play a structural role in non-mineralised cells, in the 1N-specific organic template [35]. This highlights the need for further in situ studies to better understand the function of proteins in relation to biomineralisation prior to their identification, such as in the study by Gal et al. [26] and Sakurada S. et al. [54]. Several environmental studies have begun to identify genes that may play an important role in coccolithophore biomineralisation [53,55]. These can potentially be used to identify known eukaryotic proteins involved in the processing of Ca^2+^ and CO_2_/HCO_3_^−^/CO_3_^2−^ by homology [36]. Work in this area is still very much in its infancy. The results from the Quinn [23], Kegel [55] and Gal [26] research groups would suggest that other proteins have yet to be identified and a better understanding of their role in the biomineralisation of coccolithophores is required.

After mineralisation in *P. carterae*, a coating is formed through the dissociation of PS-1 and PS-3, both of which contain coccolithosomes [51]. These polysaccharides are reportedly composed of uronic acids and sulphate esters that are attached to a polymannose main chain [58]. However, only uronic acids are considered responsible for calcite inhibition [59]. The role of organic coating on coccoliths is still relatively unclear [23], but it is hypothesised to both inhibit calcite crystal growth [24] and prevent dissolution [44,60]. It is also likely that the organic coating also gives coccoliths their superior mechanical properties; however, to the authors’ knowledge, no studies have yet measured their strength. Less attention has been paid to characterising the organic coatings on coccolithophores. Henriken et al. reported the coatings to be either an organic fibrous or granular material when studying *Coccolithus pelagicus*, *Helicosphaera carteri* and *Oolithotus fragilis*, depending on their function [30]. For example, in *Oolithotus fragilis* they reported a fibrous organic coating located at the stepped central areas of the coccoliths, providing support, and a granular organic coating on the flat surface of the coccolith.

## 4. Coccolithophore Biomineralisation

Coccolithophores offer a paradigm to ‘puzzle-solve’ the mechanisms of biomineralisation which remain poorly understood [24]. *E. huxleyi* and *P. carterae* have both been successfully grown as a model tool in climate change studies [61], indirectly answering several fundamental questions in coccolithophore biology that relate specifically to biomineralisation [24]. While these studies help explain certain observations in the biomineralisation process, many questions remain unanswered, particularly in relation to the biological control of the process [28]; questions such as, what regulates the ornate structures with such precision and uniformity? Or, why does its genetic make-up compel it to unfurl in a prescribed manner?

**Figure 3:**
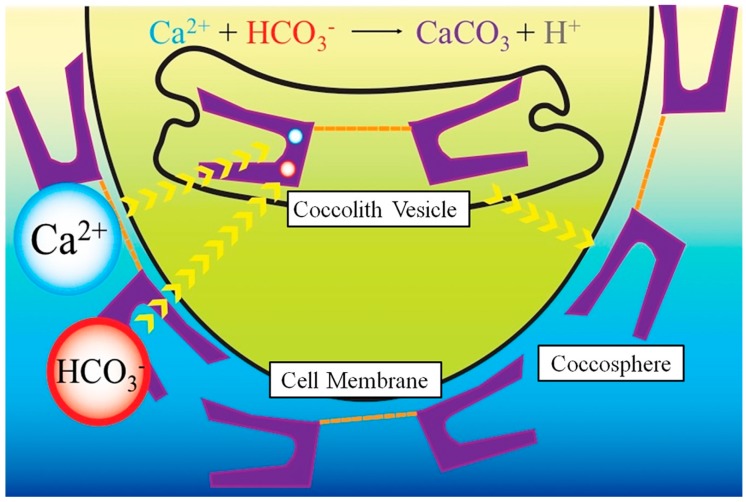
Schematic of CaCO_3_ sequestration and coccolith mineralisation for construction of coccosphere.

The first whole-genome sequence was published for *E. huxleyi* (CCMP1516) [62], providing basic insight into how these unique microorganisms evolved. In heterococcoliths, the fabrication of coccoliths occurs inside the golgi-derived coccolith-deposition vesicles, involving proteins (Section 3), lipids and an influx of Ca^2+^ from outside the cell [25]. It is noteworthy that the lipid profile of *E. huxleyi* is unusual, hosting mainly glucosylceramides, some of which contain a C9 methyl branch, which are only found in fungi and some animals [62]. The Ca^2+^ demands of coccoliths have been extensively studied in the literature by Taylor and Brownlee [22,24,52]. Ca-P reservoirs (as shown by Sviben et al. [63]) in coccolithophores are replenished through the upregulation of Ca^2+^ from external sources to prevent loss of function in a similar manner to other photosynthetic eukaryotic organisms. A critical step of their biomineralisation process is the concentrative uptake of Ca^2+^ inside the coccolith vesicle (Figure 3), while avoiding Ca^2+^ toxicity [35]. Arguably, it is the highest (*c*. 100 nmol L^−1^ Ca^2+^ ion) sustained transcellular influx of Ca^2+^ found in any cell type [24]. It is hypothesised that coccolithophores avoid toxicity by compartmentalising Ca-P reservoirs away from the coccolith vesicle-reticular body [52,63]. Ca^2+^ ions are thought to rapidly transfer directly across the membranes by diffusion during coccolith fabrication to avoid cytosol toxicity [52]. While earlier studies have eluded to their separation [35,52], Sviben S. et al. is the first study that has used state-of-the-art nanoscale imaging to show clearly that the Ca-P compartment is located adjacent to the coccolith producing compartment [63]. Further studies by Gal et al. [64] reported another compartment in addition to the Ca-P compartment, which nucleates the final calcite crystals; however, no P was observed.

The role of carbonate chemistry in the biomineralisation of coccolithophores and other phytoplankton species has been well studied [24,61,62], mainly in the context of climate change [61,65]. Bicarbonate transporters and carbonic anhydrases increase the net C fixation by concentrating CO_2_ [65]. Carbonic anhydrase could, in theory, drive coccolith formation through the generation of CO_2_ and H^+^ by causing opposite effects on the terminal domains. In spider silk formation, the polymerisation of soluble spider proteins (spidroins) and subsequent setting of fibres is hypothesised to be driven by CO_2_ and proton gradients that facilitate highly organised spatial and temporal confinement of divergent structural changes within the spinning duct [66]. Recent in vitro biomineralisation studies by Gal et al. [26] and Sakurada et al. [54] that isolate PS from the baseplate of coccoliths have reported that carbonate ions drive the biomineralisation of Ca-PS aggregates in calcite crystals.

In addition to Ca^2+^, the calcite of naturally occurring coccoliths also contains other cations like strontium (Sr^2+^), barium (Ba^2+^) magnesium (Mg^2+^) or boron (B^3+^). Recently it was suggested that the organic template of *P. carterae* demonstrates high elemental selectivity, with a strong affinity to Ca ions at the outer edge of the baseplate [49], whereas other cations (e.g., Mg) showed little or no aggregation capacity [26]. Although this review focuses solely on coccolithophores, recent work by Durak G.M. et al. [67] showed important similarities between the cytoskeleton in both calcifying (coccolithophores) and silicifying (Diatoms) haptophytes. It is clear that a full understanding of the genetic, chemical, and physical components of biomineralisation in this group of organisms is still in its infancy. Future comparative studies involving more coccolith species and relatives should reveal derived processes that will help identify general trends and approaches to biomineralisation.

## 5. Coccolithophore Source

In recent years, the morphological profiles of coccolithophores have attracted considerable interest as a marker for climate change to help predict future ocean acidification [36,39,68,69]. This has resulted in significant research into understanding aquaculture conditions to grow coccolithophores on a laboratory and semi-industrial scale [70,71]. The two most extensively studied species in lab cultures are *P. carterae* and *E. huxleyi* [10], although there are many other species [17]. Coccolithophores are generally considered particularly difficult to grow in comparison to other microalgae; however, their success rate in culture is estimated at approximately 40%, which is significantly higher than that of diatoms (15.4%) and dinoflagellates (10.6%) [72]. In general, the translation from bench to pilot scale has proven problematic and one of the main bottlenecks for all pilot microalgae cultures, particularly in the field of microalgae biofuels.

In the wild, coccolithophores are found in abundance in euphotic zones among marine phytoplankton communities. They often form huge blooms, up to 8 × 10^6^ km^2^, in which *E. huxleyi* is thought to be the predominant or only species [61,70]. They are considered the most productive calcifiers in marine habitats, accounting for approximately 50% of the global ocean calcium carbonate production [61], and they sequester approximately 10% of the carbon in the global carbon cycle [69]. A recent study by Chow et al. suggested the ability of *E. huxleyi* to form aggregates by increased production of extracellular polysaccharides and shedding of coccoliths may account for their presence in phytoplankton blooms [73]. However, the monoculture of *E. huxleyi* is not representative of the mixed population found in blooms in the wild (e.g., bacteria interactions); therefore, it is difficult to draw any major conclusions from the study, as the true response is of multiple morphotypes [34].

The reason coccolithophores create these elaborate structures is still unknown, although several hypotheses exist [25], e.g., protection against predators or ballast organic matter to help it sink onto the ocean floor [69]. Most of these hypotheses have proven unlikely [25], with the exception of its role in sequestering organic carbon [74]. Over time, coccolithophores break down and settle on the seabed, but it is unlikely that ballasting is the sole purpose for their elaborate calcified cell walls. Eventually, the coccoliths become the main component of chalk or limestone sedimentary cliffs, especially during the Cretaceous Era, which resulted in the formation of the White Cliffs of Dover, UK [75].

## 6. Translations into Strategies for Bone Repair

A critical bone defect is one that cannot self-repair. In bone tissue engineering, various biomimetic approaches [76,77] have been taken to explore solutions for repairing a critical bone defect. The principal objective in bone repair is to design a scaffold that, in most instances, will support mechanical load, facilitate vascularisation and stimulate osteoblast cell mineralisation, without invoking a chronic inflammatory response. Hydroxyapatite is the predominant natural mineral found in bone. It can be easily synthesised from calcium precursors, and has been found to have excellent biocompatibility in vivo and has been used for decades in orthopaedic applications in many different forms [78,79,80,81]. Significant challenges still exist in orthopaedics to develop suitable osteogenic material to replace conventional allograft and autograft materials [78]. The challenges in question include limited bone formation and aseptic loosening [82]. Marine materials possess naturally occurring interconnecting hierarchical structures that are advantageous to bone repair which cannot be replicated by synthetic means. Over the years, several calcite marine-based products, including ProOsteon and Algipore [7,81], have translated into commercially available bone graft products with good clinical outcomes [83]. The successful conversion of coccolithophore to hydroxyapatite has been achieved by Fee et al. [84], offering one way to translate these marine materials into bone graft materials. Coccolithophores have a much smaller particle size than the coral and mineralised microalgae that are used in the manufacture of ProOsteon and Algipore, respectively, and thus could not be used directly as a suitable bone substitute. However, they are an excellent candidate as an additive to composite materials used in bone repair. Recent advances in the field have started to place more emphasis on the importance of “*smart interfaces*”, whereby the biomaterial interface triggers a favourable cellular response [77]. Topography, particularly nano-sized surface topography, is considered to have a positive effect on cellular response [85,86]. Coccolithophores offer unique calcite surface topographies that could be of significant benefit to bone repair.

A second novel approach to translating coccolithophore biomineralisation into bone repair strategies is through protein-assisted self-assembly, which to the authors’ knowledge has, to date, not been achieved. Biomimetic synthesis using protein-assisted methods is very much in its infancy. Jain G. et al. recently successfully replicated a sea urchin spicule using rSpSM50 protein to induce spicule formation and biomineralisation using different forms of CaCO_3_ crystals [87]. Barnacle *Megabalanus rosa* (MRCP20) protein has also shown promising results in the self-assembly of calcite crystals [88]. A few studies have also shown degrees of success producing silica nanoparticles and materials using protein-assisted synthesis techniques [89,90,91]. Coccolithophore proteins offer huge potential for creating novel “*smart interface*” biomaterials for bone repair.

## 7. Conclusions and Future Directions

Advances in environmental studies using coccolithophore biomineralisation as a marker of climate change have the potential to translate into innovative nanotechnology strategies in the development of new functional materials for bone regeneration. Bone disease is a major global issue, compounded by an ageing population, modern diet and sedentary lifestyles. For example, osteoporosis causes more than 8.9 million fractures, annually, resulting in an osteoporotic fracture every 3 s [92]. The molecular control process involved in biomineralisation of marine organisms offers scientists new innovative solutions to create synthetic compounds with unique ornate structures based on the blueprints of coccolithophore, thus contribute to better healthcare solutions. For example, bio-fabrication of uniquely nano-structured, mineralised tissue scaffolds could provide implantable templates for bone regeneration. Current manufacturing strategies for such scaffolds, including 3d printing, lack the level of sophistication required to produce the nano-patterned surfaces, combined with hierarchical microstructures that are required to meet the challenges of cell signalling, guided tissue growth and optimised mechanical properties that are needed for effective bone mineral regeneration in vivo.

Further research is needed to identify proteins in the baseplate of coccolithophores and their role in the blueprints of the ornate structures produced by coccolithophores. Recent in vitro studies by Gal [3,63] and Sakurada [54] that use proteins isolated from coccolithophores as templates for synthetic calcite are paving the way towards more mechanistic studies to understand the molecular control processes in these unique structures, which could lead to the development of new nano-structured materials for bone repair.
